# The Sinuvertebral Nerve Revisited: A Morphological and Immunohistochemical Study

**DOI:** 10.3390/life16071137

**Published:** 2026-07-09

**Authors:** Lluis Aguilar, Sara Quiñones, Paloma Aragonés, Ruth Esteban-Marín, Francisco Valderrama, Marko Konschake, Maria Luque-Calvo, Clara Simón de Blas, Jose Francisco Rodríguez-Vázquez, Teresa Vázquez-Osorio

**Affiliations:** 1 Spine Unit, Hospital Clínic de Barcelona, 08036 Barcelona, Spain; laguilar8762@gmail.com; 2 Spineli, Unitat de Columna, Clinica Corachan, 08017 Barcelona, Spain; 3 Patology Service, La Paz Hospital, 28046 Madrid, Spain; saraquga97@gmail.com; 4 Surgery Department, School of Medicine, Complutense University of Madrid, 28040 Madrid, Spain; paloarag@ucm.es; 5 Body Donation Centre and Dissection Rooms, Complutense University of Madrid, 28040 Madrid, Spain; fvalderr@ucm.es (F.V.); jfrodvaz@ucm.es (J.F.R.-V.); 6 Human Anatomy and Histology Department, Zaragoza University, 50009 Zaragoza, Spain; ruthesteban@unizar.es; 7 Anatomy and Embryology Department, School of Medicine, Complutense University of Madrid, 28040 Madrid, Spain; 8 Department of Anatomy, Histology and Embryology, Medical University of Innsbruck, 6020 Innsbruck, Austria; marko.konschake@i-med.ac.at (M.K.); maria.luque@i-med.ac.at (M.L.-C.); 9 Department of Operative Research and Statistics, Rey Juan Carlos University, 28933 Madrid, Spain; clara.simon@urjc.es

**Keywords:** sinuvertebral nerve, low back pain, discogenic lumbar disease, lumbar plexus, spinal nerve

## Abstract

The proven involvement of the sinuvertebral nerve (SVN) in discogenic low-back pain and the demonstration that its blockade has been effective in reducing the intensity and frequency of diffuse low back pain have led to an increase in publications related to the characterization of this nerve. However, there is a huge disparity in the observations resulting from the studies carried out, probably due to the technical difficulty of accessing this structure. In recent years, the number of studies in large samples has increased but some important data in relation to the nature of the sinuvertebral nerve remain unpublished. We studied 100 vertebral column segments between L1 and L5, corresponding to both sides of 10 adult cadavers donated to the Body Donation Centre and Dissection Rooms of the Complutense University of Madrid. All levels were carefully dissected to study sinuvertebral nerve origins and some samples of SVN were selected to be routinely paraffin-embedded and serially sectioned with a Minot-type microtome at a 7 µm thickness. Immediately after dewaxing following the standard histology lab protocols, sections from the selected SVN (well-preserved morphology and histologic condition) were subjected to an immunohistochemical protocol to detect CGRP-IH and VIP-IH. Data analysis was performed using IBM SPSS Statistics version 27 and RStudio. The SVN was observed with a single branch (pattern I) in 82 cases (85.4%) and with two branches (pattern II) at the same level in 14 cases (14.6%). Statistical differences were not found in relation to vertebral levels, side or sex. All sinuvertebral nerve samples that underwent immunohistochemical study were positive for CGRP and VIP, suggesting a mixed autonomic (VIP+) and sensory (CGRP+) fibre composition along the nerve trunk. This study confirms the neurochemistry profile of the SVN due to the realization of the immunochemistry characterization directly in the SVN, not in its innervated structures. This information supports the usage of SVN blocking from a pathophysiological point of view for diagnostic and treatment techniques (e.g., Percutaneous Transforaminal Endoscopic Radiofrequency Ablation of the SVN) in discogenic lumbar pain.

## 1. Introduction

The proven involvement of the sinuvertebral nerve (SVN) in discogenic low-back pain [1,2,3,4,5,6,7,8] and the demonstration that its blockade has been effective in reducing the intensity and frequency of diffuse low back pain [8,9,10] have led to an increase in publications related to the characterization of this nerve [11,12].

The first thing that stands out after analyzing the publications related to the sinuvertebral nerve is the disparity in the observations resulting from the studies carried out, probably due to the technical difficulty of accessing this structure. Studies in human cadaveric material began with small samples [1,2,4,5,6,13,14,15,16,17,18] and continued with larger sample sizes [11,19] which, however, have not yielded more homogeneous results.

Despite efforts to further its study, considerable controversy remains regarding the origin, course, and distribution of the nerve. Most authors define the origin as a neural branch emerging from the spinal nerve and a postganglionic sympathetic branch developing from the communicating branch [1,4,5,6,7,15,20]. Other authors define a single origin as a spinal branch [1,5] or an exclusively sympathetic branch [15,21,22]. Its course is described as ascending branches [1,4,5,18], descending branches [1,4,5,7], oblique horizontal branches [6,7], and mixed branches dividing into a descending and an ascending branch [4,5,20,21,22,23,24], although plexiform patterns have also been described [12,21,22,23,24].

The SVN has been described as forming ipsilateral [4,5,21] or contralateral [4,5,21,22,23,24] connections, or showing no connections [1,11,15,18,20].

Regarding its immunohistochemistry, even today there are profound discrepancies in the precise somatic, sympathetic, or mixed composition of the sinuvertebral nerve [18,21,22,23,25]. Some authors have performed immunohistochemical studies with the sole purpose of determining the nervous nature of the branches they observed and thus being able to consider them as deputy branches of the sinuvertebral nerve [12]. The histological characterization of the sinuvertebral nerve has also been proposed in various studies to discern its sensory, motor, and/or autonomic nature, and even as a confirmatory method for identifying structures that could initially be described as sinuvertebral nerves [19,26,27].

While previous studies have successfully mapped a variety of neuropeptides at the nerve endings within its innervated target structures—such as the intervertebral disc and the posterior longitudinal ligament—the intrinsic neurochemical profile directly on the isolated nerve trunk itself remains completely unverified [2,4,5,6,13,14,15,18]. Performing immunohistochemistry directly on the main nerve trunk represents a highly novel approach, as it definitively isolates its true functional nature from surrounding tissue contamination and avoids the confounding signal noise inherent to peripheral targets. This study aims to systematically characterize the neurochemical composition of the lumbar SVN. We hypothesize that the human lumbar SVN trunk contains a mixed population of nerve fibres exhibiting both VIP and CGRP immunoreactivity, which would be consistent with a complex autonomic and sensory structural composition.

## 2. Material and Methods

We studied 100 vertebral column segments between L1 and L5, corresponding to both sides of 10 adult cadavers donated to the Body Donation Centre and Dissection Rooms of the Complutense University of Madrid. Five were male and five were female, aged between 53 and 94 years, all preserved in a solution containing ethanol, formaldehyde, phenol, and glycerin for a minimum fixation period of 6 months. Subsequently, those branches that appeared thicker at different levels were cut and fixed in 4% paraformaldehyde for 24 h at 4 °C for immunohistochemical testing.

All specimens were obtained through a voluntary body donation programme. We strictly adhere to Spanish legislation regarding anatomical donation, medical research, and data protection. This program operates in rigorous compliance with the regulatory framework governing the use of anatomical material and clinical data, including Decree 2263/1974 (Reglamento de Policía Sanitaria Mortuoria), the foundational Law of 18 December 1950 (Obtención de piezas anatómicas para injertos), Law 41/2002 (Autonomía del Paciente) for informed consent and clinical records, and Organic Law 3/2018 (LOPDGDD) for data privacy. Explicit, written informed consent was obtained from every donor prior to death. All handled donor data have been strictly pseudonymized to maintain absolute confidentiality.

After removing the dorsal musculature, neural arch, dura mater, and spinal cord, we exposed the anterior vertebral plexus, spinal artery, and sinuvertebral nerve. The dissection was performed using a Zeiss surgical microscope (Carl Zeiss AG, Oberkochen, Germany) (0.6× to 2.5×).

The lumbar sympathetic trunk was identified and dissected along its length, including (as far as magnification allowed) all its branches. Visceral and aortic branches were not studied. All branches passing around the vertebral column were dissected as far as possible along their course. Communicating rami were marked from their junction with the ventral primary rami of the spinal nerves.

For the identification and anatomical dissection of the ventral spinal nerves, the sympathetic chains and spinal nerves of the lumbar spine were first identified.

Next, microdissection of the selected segments was performed to locate the ventral spinal nerve roots of origin and their initial course. Once the sympathetic chains and lumbar roots were identified, the lumbar spine was cut, and the posterior arches were removed at the level of the pedicles. Using microdissection, the recurrent branches on each side were identified. Subsequently, those branches that appeared thicker at different levels were cut and fixed in 4% paraformaldehyde for immunohistochemical testing.

The SVNs were routinely paraffin-embedded and serially sectioned with a Minot-type microtome at a 7 µm thickness. Immediately after dewaxing following the standard histology lab protocols, sections from selected SVN (well-preserved morphology and histologic condition) were subjected to an immunohistochemical protocol to detect CGRP-IH, and VIP-IH. The staining of the antibodies CGRP (mouse monoclonal, Abcam 4901) (Abcam, Cambridge, UK) and VIP (rabbit polyclonal, Boster RP1108) (Boster Biological Technology, Pleasanton, CA, USA) was done onto a Ventana Discovery Ultra^®^ (Roche Diagnostics, Mannheim, Germany) as following: sections were pre-treated for 32 min (VIP), or 48 min (CGRP), at 97 °C with a pH 8.5 Tris-Borat-EDTA buffer (Ventana, 950-500) (Ventana Medical Systems, Inc., Tucson, AZ, USA). Prior to primary antibody incubation, the endogen peroxidase was blocked for 12 min in Dic Inhibitor (Ventana^®^ 760-4840). Sections where incubated either in VIP serum (final dilution 1 µg/mL; 1 h at 37 °C) or CGRP serum (final dilution 2 µg/mL; 2 h at RT). For the signal detection the Ultravision LP Detection Kit (Labvision, TL-060-HL) (Lab Vision Corp., Fremont, CA, USA) and the ImmPACT™ DAB EqV Peroxidase Substrate (VectorLabs, 4103-100) (Vector Laboratories, Newark, CA, USA) were used.

Positive controls where developed, with the same protocol, in human colon (CGRP) and pancreas (VIP).

Inmunohistochemical controls:

Immunohistochemical validation was performed using external human positive controls, specifically colon tissue for CGRP and pancreas tissue for VIP. Separate negative reagent controls (omission of the primary antibody) were not conducted. Instead, tissue specificity was continuously monitored via internal negative controls within each analyzed spinal nerve section; non-target structures, including the connective tissue of the epineurium and adjacent vascular walls, consistently demonstrated a complete absence of immunoreactivity, confirming the high specificity of the primary antibodies and the automated detection system (Ventana Discovery Ultra^®^) (Roche Tissue Diagnostics, Ventana Medical Systems, Inc., Tucson, AZ, USA).

Sections were selected for immunohistochemical processing based on strict semi-quantitative criteria for well-preserved morphology. Specifically, tissue integrity was defined by the complete retention of nerve trunk epineurium, absence of extensive tissue fragmentation or detachment from the slide, and minimal post-mortem autolysis artefact under light microscopy examination. Any sample failing to meet these baseline microstructural criteria was discarded prior to staining.

Due to the inherent nature of gross spinal microdissection, investigators could not be blinded to the vertebral level or anatomical side during the initial tissue-harvesting phase. However, to prevent bias during histological evaluation, complete blinding was strictly implemented for the subsequent microscopic analysis. All immunohistochemical slides were assigned alphanumeric codes by an independent laboratory member. The two investigators evaluating fibre immunoreactivity and composition remained completely blinded to the vertebral level, side, sex, and age of the source specimens until all data collection was finalized.

A sample size justification was performed based on the expected prevalence of the SVN from historical anatomical data (>90%). Investigating a total of 100 segments guarantees a margin of error below 5% at a 95% confidence level, ensuring the generalizability of the findings. Dissection integrity was verified for each segment; any level exhibiting irreversible mechanical or structural damage during tissue preparation was strictly excluded a priori from the final morphometric and pattern analysis to prevent exclusion bias.

Statistical analysis:

Data analysis was performed using IBM SPSS Statistics version 27 and RStudio (RStudio software version 2026.06.0; Posit Software, PBC, Boston, MA, USA). For the comparison analysis by side, type and lumbar region, Chi-squared and Anova tables were considered. Statistical significance was defined using a stringent alpha level of 0.005 to reduce type I errors of false positives. The strength and direction of the relationship among variables was evaluated by means of the non-parametric Spearman Correlation statistic. To evaluate the strength of the association between multiple independent variables, multiple correspondence analysis was considered.

## 3. Results

Ten vertebral columns were dissected between segments L1 and L5, constituting an initial sample of 50 levels on both sides (left and right). Out of these, four levels were excluded prior to analysis due to procedural microdissection issues (uncontrollable tearing during the removal of high-calibre vertebral veins), yielding a final sample size of N = 96 levels for evaluation. The presence of the sinuvertebral nerve was successfully confirmed in 100% of these analyzed levels (n = 96).

In all cases, the SVN originated from the union of a somatic root from the corresponding spinal nerve and a sympathetic branch from the communicating ramus. After its origin, the nerve followed a recurrent course to enter the intervertebral canal.

The SVN was observed with a single branch (pattern I) in 82 cases (85.4%) and with two branches (pattern II) at the same level in 14 cases (14.6%).

**Figure 1:**
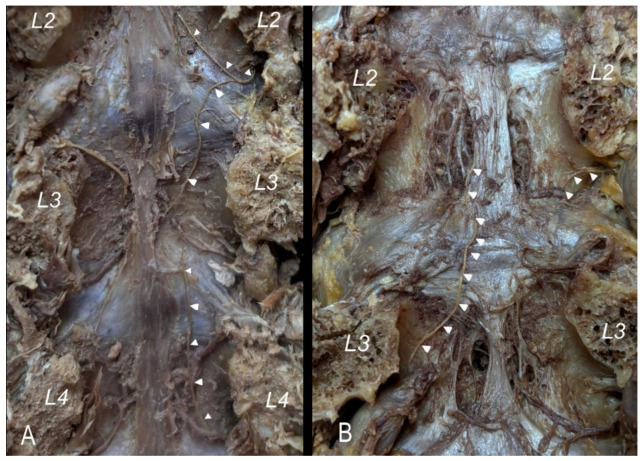
Vertebral canal posterior views showing pattern 1. Sinuvertebral branches labelled with white arrow heads. Vertebral pedicles are numbered. (**A**) Type 1a. One right single sinuvertebral nerve ascends from L4 to L3 and type 1b right L2 sinuvertebral nerve divided into two branches, one ascending and one descending terminating at the midline. (**B**) Type 1c a left single branch arises from L3 level and ascends two levels above its entry into the intervertebral canal (L2 vertebral body) and type 1d a single right descending L2 sinuvertebral nerve.

The pattern I cases presented variable arrangements, with four distinct situations classified into four types (Figure 1):

Type 1a: The sinuvertebral nerve ascends to approximately the middle third of the vertebral body of the vertebra immediately above, covered by the posterior longitudinal ligament. This situation was observed in 64.6% of cases (n = 62).

Type 1b: The sinuvertebral nerve divided into two branches, one ascending and one descending, terminating at the midline. This situation was observed in 17 cases (17.7%).

Type 1c: In two cases (2.1%), a single branch ascended, terminating two levels above its entry into the intervertebral canal.

Type 1d: In one case (1%), the sinuvertebral nerve showed a descending course.

**Figure 2:**
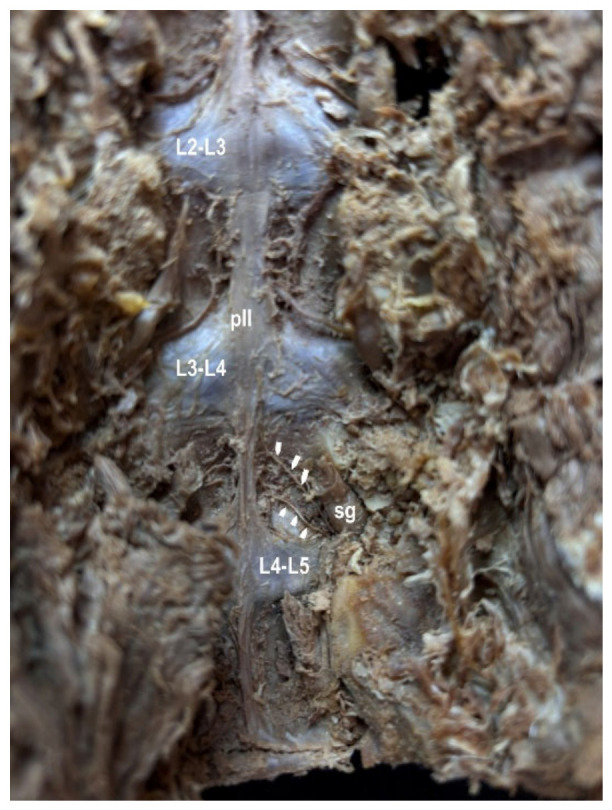
Vertebral canal posterior view showing a double sinuvertebral nerve in the right side. (pattern 2). Both branches are labelled with white arrow heads. Intervertebral discs are numbered. (pll) Posterior longitudinal ligament, (sg) spinal ganglion.

Only one disposition was observed in cases of patten 2 (Figure 2).

The different patterns and types were assessed according to the vertebral level, observing a homogeneous distribution at all levels. Statistical differences were not found in relation to vertebral levels (Table 1).

Table 2 shows the degree of association between the different types according to Spearman’s correlation. A negative association was only found between the L3 and L4 vertebrae (rho = −0.623; *p*-value = 0.003). No significant correlation was found between the side and the types considered without taking the lumbar vertebra into account (Spearman’s rho = −0.068; *p*-value = 0.503).

**Figure 3:**
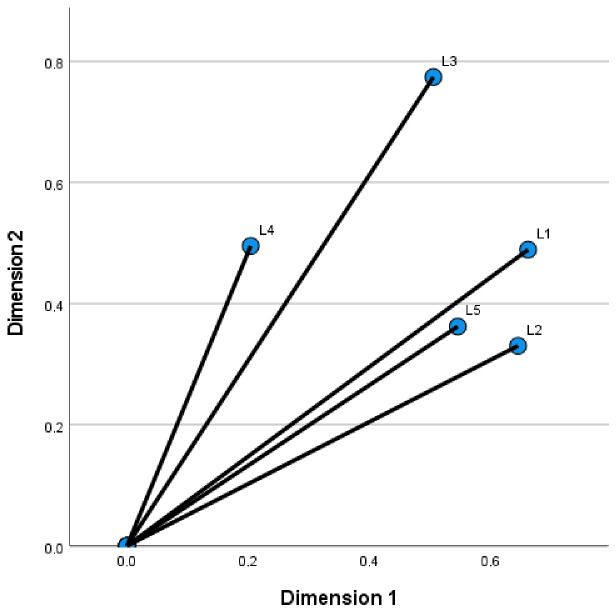
Bispatial diagram of multiple correspondence analysis according to the type considered and the lumbar vertebrae.

A multiple correspondence study was conducted to examine the association of the different types considered according to the lumbar vertebrae. The results show a Cronbach’s alpha of 0.762 for the first dimension and 0.740 for the second dimensions (resulting in a high degree of association). The first component grouped lumbar vertebrae L1, L2, and L5. The second component grouped lumbar vertebrae L3 and L4 (Figure 3).

Ipsilateral nerve connections were observed in 31 cases at different levels. No statistical differences were observed by level, side or sex.

**Figure 4:**
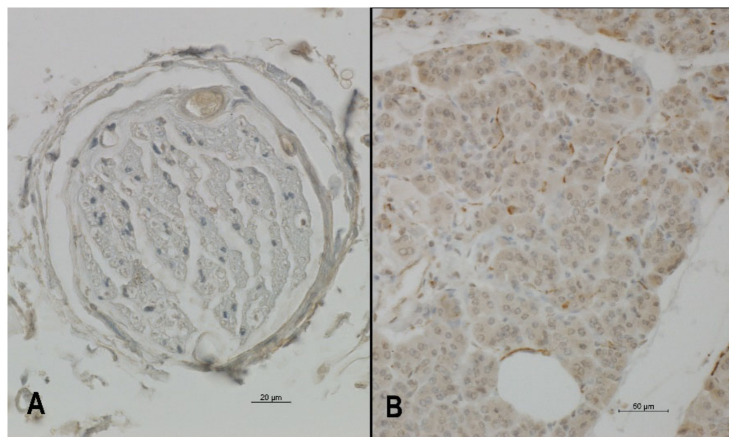
(**A**) Immunohistochemical localization of VIP within the human lumbar sinuvertebral nerve trunk. Dense VIP-positive chromogen precipitation is confined to the cytoplasmic matrix and elongating processes of Schwann cells (arrowheads) enveloping unmyelinated axons. Intranuclear spaces remain strictly negative, matching the secretory profile of the neuropeptide. (**B**) Positive control of VIP made in pancreas (20×).

**Figure 5:**
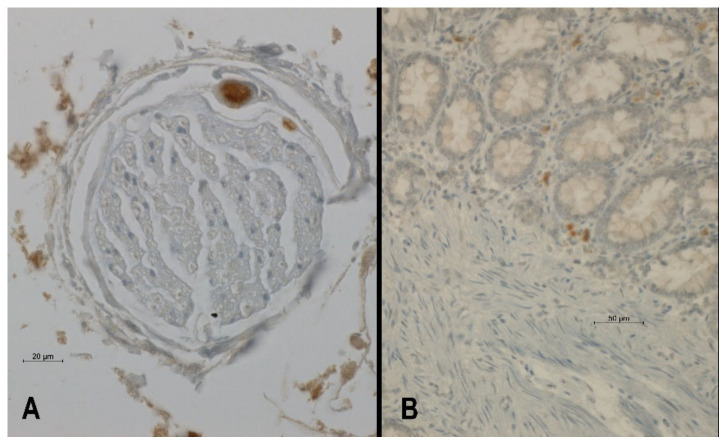
(**A**) Immunohistochemical localization of CGRP within the human lumbar sinuvertebral nerve trunk. Micrographs show intense CGRP-immunoreactivity localized within the axoplasm of fine nerve fibres and the perinuclear cytoplasm of associated Schwann cells. Note the complete absence of staining within the connective tissue epineurium. No true nuclear accumulation is observed. (**B**) Positive control of CGRP made in colon (20×).

All sinuvertebral nerve samples that underwent immunohistochemical study were positive for CGRP and VIP, suggesting a mixed autonomic (VIP+) and sensory (CGRP+) fibre composition along the nerve trunk (Figure 4 and Figure 5).

## 4. Discussion

Regarding its origin, several theories have been described that consider an exclusively sympathetic origin [18,21] or a mixed origin in some cases [5,15] or in all cases [4,6,7,11,19,20]. In 100% of cases, we observed a mixed origin (sympathetic and spinal) for the sinuvertebral nerve, as reported by other authors and as observed in previous studies made by our laboratory [19].

The difficulty of the dissection technique, as well as the fact that the nature of the observed nerves has not been histologically verified, may explain these differences [19,26].

Our immunohistochemical analysis reveals that the human lumbar sinuvertebral nerve trunk exhibits consistent VIP-immunoreactivity (VIP-ir) and CGRP-immunoreactivity (CGRP-ir). While VIP is not exclusively sympathetic and can be found in other autonomic or sensory pathways, and CGRP mediates broader sensory and vasodilatory functions rather than purely nociceptive transmission, their combined presence is consistent with a mixed autonomic and sensory fibre composition. These findings suggest a complex, dual structural architecture within the nerve trunk, though they do not provide definitive functional classification of individual fibre subtypes.

The distribution of the different patterns and types is homogeneous, with no significant differences by vertebral level, side, or sex. These results are consistent with those observed by most authors but differ from those published by Raoul et al. [18], who describe a variable distribution depending on the vertebral level and a greater number of sinuvertebral nerve branches at L2. Regarding this level (L2), we found type 1a distributions more frequently, but this difference was not statistically significant as has been shown in Table 1.

Connections between sinuvertebral nerves at adjacent levels have also been classically described. In our study, we observed this in 32% of cases, always ipsilaterally. We did not observe any branches crossing the midline, although it is worth reiterating the difficulty of the dissection technique in these very small structures, which could explain the differences.

Many authors have studied the immunochemical characterization based on the structures innervated by the SVN, such as the posterior surface of intervertebral disc (IVD), posterior longitudinal ligament and anterior aspect of meninges [2,4,5,6,13,14,15,18,21,28,29,30,31,32,33].

In the case of the human IVD, the immunohistochemical characterization of these fibres’ endings have yielded the presence of a variety of neuropeptides involved in nociceptive neurotransmission, PGP 9.5, SP, CGR, NPY, and VIP [16,17,18,34,35,36,37,38].

In addition, in degenerated human IVD, from patients with clinical symptoms, there is an extensive spread of vessels and nerve fibres even reaching the nucleus pulposus [39,40], that correlates with the expression of nerve growth factor [41] and inflammatory markers such as tumour necrosis factor-alpha [42].

In the case of annulus fibrosus, some results have shown immunoreactivity to general nerve markers (synaptophysin gene product and protein 9.5) and to neuropeptides (substance P and C terminal flanking peptide of neuropeptide Y) [17].

Previous observations have found in the posterior longitudinal ligament the presence of different nervous endings containing substance P, and occasionally finding the presence of enkephalin+, indicating modulation of pain signals [43].

Other authors have based their research in fetuses, for example Groen et al. (1990) with a choline acetyl esterase staining, having as a main limitation the inability to determine if these patterns are maintained within the adult population [33].

From a clinical and neurosurgical perspective, establishing a robust neurochemical and morphological profile of the human lumbar SVN trunk is vital for resolving the widespread diagnostic mismatch frequently observed between lumbar spine neuroimaging and a patient’s subjective complaints. Incidental radiological findings, such as lumbar facet joint abnormalities, ligamentous cysts, or even rare developmental remnants like cystic dilation of the ventriculus terminalis (CDVT), are frequently uncovered on routine MRI exams. As highlighted by Ganau et al. (2012) [44] in their updated classification of CDVT, patients categorized under Type 1a exhibit nonspecific symptoms that do not mechanically correlate with the central lesion itself. In these complex clinical scenarios, localized entrapment, microvascular engorgement, or peptidergic inflammation of the mixed autonomic-sensory SVN trunk may serve as the true underlying source of axial discomfort. Recognizing the consistent distribution of nociceptive and autonomic pathways along the SVN trunk can help operating spine surgeons avoid aggressive, unnecessary central spinal procedures, steering the management plan toward targeted diagnostic nerve blocks or conservative therapies instead.

Some studies have also suggested that the terminal branches of the SVN could act as mechanoreceptors, pressure receptors, and nociceptors or thermal terminals [14]. Therefore, even today, in the case of the SVN and its neurochemical profile there are still profound discrepancies in the somatic, sympathetic, or mixed composition. For this reason, the present study confirms the neurochemistry profile of the SVN thanks due to the realization of the immunochemistry characterization directly in the SVN, not in its innervated structures. This information supports the usage of the SVN blocking from a pathophysiological point of view for diagnostic and treatment techniques (e.g., Percutaneous Transforaminal Endoscopic Radiofrequency Ablation of the SVN) in discogenic lumbar pain [8,10].

Finally, a minor limitation of this study includes the lack of dedicated slide-omission negative controls during the immunohistochemical runs; however, the absolute lack of staining in the internal connective and vascular tissues within the nerve trunk strongly validates the specificity of our neurochemical findings.

## Figures and Tables

**Table 1:** Frequency of each of the patterns and types at each of the vertebral levels studied.

Type	L1	L2	L3	L4	L5
Pattern 1/Type 1a	12	14	12	12	12
Pattern 1/Type 1b	5	3	5	2	2
Pattern 1/Type 1c	1	0	1	0	0
Pattern 1/Type 1d	0	0	0	0	1
Pattern 2	2	3	1	4	4

Chi-square test of independence shows no significant differences by side in any of the lumbar vertebrae (L1: $\chi_{3}^{2}$ = 1.53; *p*-value = 0.675; L2: $\chi_{2}^{2}$ = 0.667; *p*-value = 0.717; L3: $\chi_{4}^{2}$ = 3.533; *p*-value = 0.473; L4: $\chi_{3}^{2}$ = 2.33; *p*-value = 0.506; L5: $\chi_{4}^{2}$ = 2.33; *p*-value = 0.675). If the study is considered independently of the lumbar vertebra, no significant differences are found according to the side either. ($\chi_{5}^{2}$ = 4.603; *p*-value = 0.466).

**Table 2:** Spearman’s correlation between vertebrae according to the types considered.

	L1	L2	L3	L4	L5
L1	1.000	0.348	0.056	−0.204	0.372
L2	0.348	1.000	−0.204	0.041	0.128
L3	0.056	−0.204	1.000	−0.623 **	0.009
L4	−0.204	0.041	−0.623 **	1.000	−0.271
L5	0.372	0.128	0.009	−0.271	1.000

** The correlation is significant at the 0.01 level (two-tailed).

## Data Availability

The original contributions presented in this study are included in the article. Further inquiries can be directed to the corresponding author.
